# Phytic Acid Intercalated Graphene Oxide for Anticorrosive Reinforcement of Waterborne Epoxy Resin Coating

**DOI:** 10.3390/polym11121950

**Published:** 2019-11-27

**Authors:** Na Wang, Huiying Gao, Jing Zhang, Ye Qin, Deyi Wang

**Affiliations:** 1 Sino-Spanish Advanced Materials Institute, Shenyang University of Chemical Technology, Shenyang 110142, China; ghy724@163.com (H.G.); zhangjingcszx@syuct.edu.cn (J.Z.); 2 Advanced Manufacturing Institute of Polymer Industry (AMIPI), Shenyang University of Chemical Technology, Shenyang 110142, China; 3 IMDEA Materials Institute, C/Eric Kandel 2, Getafe, 28906 Madrid, Spain; deyi.wang@imdea.org

**Keywords:** waterborne epoxy coating, electrochemical impedance spectroscopy, anticorrosion, phytic acid, graphite oxide

## Abstract

Epoxy resin coatings were prepared with phytic acid-doped graphene oxide (PA-GO) to modify epoxy resins (EP). The aim was to improve the dispersion of GO in waterborne epoxy resin, and thus to improve the corrosion resistance of steel structures. The Fourier transform infrared spectroscopy (FTIR), X-ray diffraction (XRD), and X-ray photoelectron spectroscopy (XPS) results showed that PA-GO was successfully prepared and has a better dispersion in epoxy resin. This is mainly due to the PA insert, which increased the layer spacing of the GO. The results obtained under the controlled corrosive environment showed that the specimen coated with EP containing 1.0 wt.% PA-GO had better corrosion resistance than other samples. This resistance was also two orders of magnitude higher than pure epoxy coating. The main reason for this is that the dispersion of GO in waterborne epoxy resin had been improved.

## 1. Introduction

Epoxy resins (EP) have many excellent chemical properties and adhere to various substrates that are widely used in metal structure/surface anticorrosion [1,2]. However, at present, epoxy resins commonly used for corrosion protection are typical solvent-based systems containing extremely high volatile compounds (VOC), which are extremely harmful to the environment and human health. Therefore, the use of epoxy resins must shift from solvent-based systems to aqueous systems due to environmental and human health considerations and national legislation [3,4,5]. However, waterborne epoxy resin coating has the disadvantages of poor barrier performance and a short anti-corrosion period due to the easy formation of micropores during curing in practical application [6,7,8]. Therefore, the barrier property of waterborne epoxy resin is often improved by adding functional fillers (nanocarbons [6], SiO_2_ [9], and ZnO [10]) with excellent performance to improve the anti-corrosion performance of the coating and prolong the anti-corrosion period.

In our previous work, water-based composite epoxy coatings with good barrier performance and corrosion resistance were developed with layered sodium montmorillonite (Na-MMT) [11], mesoporous MCM-41 silica nanoparticles [12], polyethylene imine (PEI)-modified meso-TiO_2_ [13], and tripolyphosphate intercalated hydrotalcite as fillers [14]. The results show that these functional fillers can inhibit the corrosion process in the coating. For example, sodium tripolyphosphate intercalated hydrotalcite improves the solubility of hydrotalcite and increases the compatibility of filler with water-based epoxy resins, thus effectively impeding the transport of corrosive electrolytes through the coating [14]. Therefore, layered materials and materials with channel structures can improve the corrosion resistance of waterborne epoxy resin.

Graphene oxide (GO) is a type of two-dimensional (2D) layer structure of nanomaterials with excellent blocking performance that can be widely applied in many fields [15,16,17,18,19,20]. GO also has important applications in the field of coating corrosion protection [21,22,23]. Wu et al. prepared a Mg(OH)_2_/GO composite film that was electrochemically deposited on AZ91D magnesium alloys with a constant potential to delay material corrosion [24]. Fayyad, Eman M. et al. prepared oleic acid grafted chitosan/GO composite coatings to enhance the corrosion resistance of the coatings by improving the hydrophobicity of the materials [25]. Parhizkar, Nafise et al. prepared a sol-gel-based silane coating filled with amino and isocyanate silane functionalized graphene oxide nanosheets for low carbon steel corrosion protection [26]. These findings suggested that graphene oxide has an important role in the field of anti-corrosion. Our previous work involved the modification of GO in water-based epoxy resin. We grafted ZSM-5 onto the GO surface to improve the anti-corrosion effect of GO. The results show that this is mainly due to the synergistic effect of the hole/sheet structure to improve the anti-corrosion performance of the coating [27]. However, GO is used in water-based epoxy resins to solve the problems of interlayer forces, water solubility, and dispersion. 

Phytic acid (PA) has good solubility in water. Given its wide availability, unique structure, and chelating properties, it has also been studied as an environmental protection corrosion inhibitor for many metals [28]. Therefore, we want to modify GO with phytic acid, which can dissolve in water and chelate metal ions, to improve the dispersion of GO in water-based epoxy resin and to improve the anticorrosive performance of the coating.

In this paper, PA-GO composites were prepared by chemical doping GO with phytic acid. PA-GO and GO were filled into the EP to prepare epoxy resin coatings to improve the barrier performance and corrosion resistance of epoxy coating. The dispersion of the two in water-borne epoxy resin was investigated, and the corrosion resistance of EP, GO/EP and PA-GO/EP was evaluated in a NaCl solution-controlled environment.

## 2. Materials and Methods 

### 2.1. Materials

Sodium hydroxide, sodium nitrite, phytic acid (PA), and potassium permanganate were obtained from Tianjin Damao Chemical Reagent Factory (Tianjin, China). Anhydrous ethanol was obtained from Tianjin Yongda Chemical Reagent Co., Ltd. (Tianjin, China). Hydrogen peroxide, sodium hydroxide and ammonia were obtained from Liaoning Jiacheng Fine Chemicals Co., Ltd. (Fuxin, China). Sulfuric acid (98%), graphite, and *N,N*-dimethyl acetamide (NMF) were obtained from Tianjin Ruijinte Chemical Co., Ltd. (Shenyang, China). Waterborne epoxy resin and curing agent were obtained from Hexion Specialty Chemicals, Inc. (Columbus, OH, USA).

### 2.2. Synthesis of PA–GO

GO was obtained by oxidation of natural graphite powder according to the modified Hummers’ method [29]. To begin with, 4 g sodium hydroxide was dissolved in 100 mL solvent to form a homogeneous solution (denoted as the solution A, the volume ratio of H_2_O to C_2_H_5_OH in the solvent is 1:1) at ambient temperature. Subsequently, solution B was prepared by dissolving 2.9 mL 70% phytic acid in 15 mL 1% acetic acid at ambient temperature. All experiments were performed in a three-necked flask at 35 °C. First, 0.8 g GO was added to 100 mL water, and the mixture was subject to ultrasonic shock for 30 min. Then, 100 mL GO suspension and 1 mL ammonia were added to the flask and stirred for 30 min. Then, solution B was added and stirred for 1 h. Solution A was subsequently added. After the reaction was completed, 100 mL C_2_H_5_OH was added to the mixed solution, and a black gelatinous substance was obtained. The black gelatinous substance was dissolved in *N,N*-dimethyl formamide solution (DMF, 60 mL) under sonication and stirred for 24 h. Finally, the substance was washed in deionized water 6–8 times. After freeze-drying, the product obtained was named PA-GO.

### 2.3. Preparation of the Composite Coatings

Composite coatings were prepared using waterborne epoxy resin with fillers of PA-GO (at various quantities and weight percentages as shown in Table 1). After milling, the curing agent and deionized water were added into the mixture. Afterwards, the mixture was stirred for another 20 min (at 120 ppm by the magnetic stirrer) to yield the final composite coating. In this work, the parent coating (i.e., the neat epoxy) and the composite coating with the GO filler were also prepared as the reference materials for comparison (Table 1).

To prepare the surface of the steel substrate (with rounded corners/edges), steel substrates were first polished using fine emery paper, washed with acetone, and dried at 40 °C for further use. The prepared steel substrates were painted using a compressed air sprayer (using the samples in Table 1) and cured at room temperature. After the solidification treatment of the paint-coated steel substrates, the thickness of the coating was measured using a Qnix4500 digital meter (Nix, Germany), which revealed that the average coating thickness is 50 ± 5 µm. The final-coated specimens were stored in a desiccator for a week before any tests.

### 2.4. Characterization of Materials 

Fourier transform infrared spectroscopy (FTIR) was performed on a Nicolet MNGNA-IR560 instrument (Artisan Technology Group, Austin, TX, USA) with 4 cm^−1^ resolution. Thermalgravimetric analysis (TGA) was conducted under nitrogen (N_2_) flow (heating rate = 10 °C/min) on a NETCH STA449C thermogravimetric analyzer (NETZSCH, Selb, Germany). X-ray diffraction patterns were recorded on D/max-2500PC X-ray diffractometer (Rigaku, Tokyo, Japan) using CuKα radiation at 50 kV and 200 mA with a scanning rate of 2.4° min^−1^ and 0.05 steps. The morphology of GO and PA-GO was examined with scanning electron microscopy (SEM) using a JSM-6360LV SEM (JEOL, Tokyo, Japan). XPS spectra were obtained using a VG ESCALAB MKLL electron spectra meter (London, UK) equipped with an Al Ka X-ray source.

Electrochemical impedance spectroscopy (EIS) of coatings was performed using a Metrohm electrochemical workstation (Autolab 84362 with ZSimpwin software, Metrohm, Shanghai, China) to verify the effect of the developed fillers on the anticorrosion performance of epoxy coating (50 ± 5 μm thickness, 9.6 cm^2^ measurement area) on the mild steel substrate. The impedance measurement was performed at room temperature in 3.5 wt.% NaCl solution, and the experimental data were normalized with respect to 1 cm^2^. Under the open circuit potential, the test system consisted of a three-electrode cell composed of a saturated calomel electrode (SCE, as the reference), a platinum electrode (as the counter) and a coated coupon (as the working electrode). Repeated measurements were conducted (at least three times) with the coatings used in this work, demonstrating that the values were reproducibly better than ±2%. Impedance spectra of coupons after different immersion times were recorded in a frequency range of 10^−1^ to 10^5^ Hz with the sinusoidal alternating potential signal of 10 mV.

The corrosion test of the coated specimens was also performed by the neutral salt spray test. The condition used by the salt spray apparatus (YWα/Q-150) was specified by ASTM B117 [30], i.e., continuous spraying with 5.0 wt.% NaCl solution at 35 ± 2 °C. The surface of the test specimens was inspected, and the degree of rusting was rated by visual examination of the test specimens after 500 h. 

## 3. Results and Discussion

### 3.1. Characterization of GO and PA-GO 

**Figure 1:**
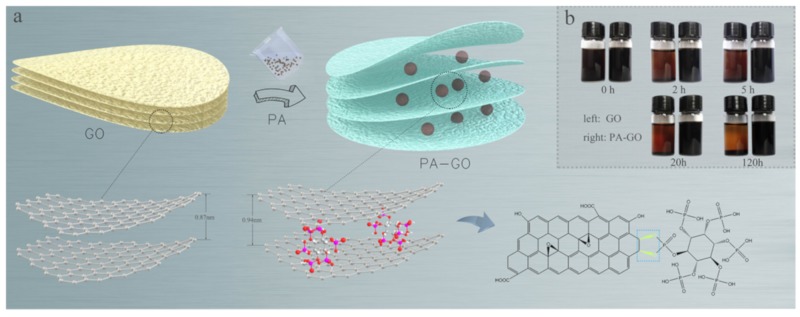
The possible reactions during PA intercalation of GO. (**a**) The reaction of PA and GO, (**b**) and the dispersion of GO (left) and PA-GO (right) in water after various storage periods.

Figure 1 shows the possible reactions during PA intercalation of GO. PA covalently bonds with the epoxy group on the GO surface to form a functional filler. The sedimentation test in Figure 1b shows the dispersive ability of PA-GO and GO in an aqueous solution. In this experiment, GO and PA-GO were dispersed in water by ultrasonic dispersion for 0.5 h, and then the dispersion was stored without interference for numerous examinations. As shown in Figure 1b, after 5 h, no obvious stratification occurred in the PA-GO solution, indicating that PA modified GO had better dispersion in water.

**Figure 2:**
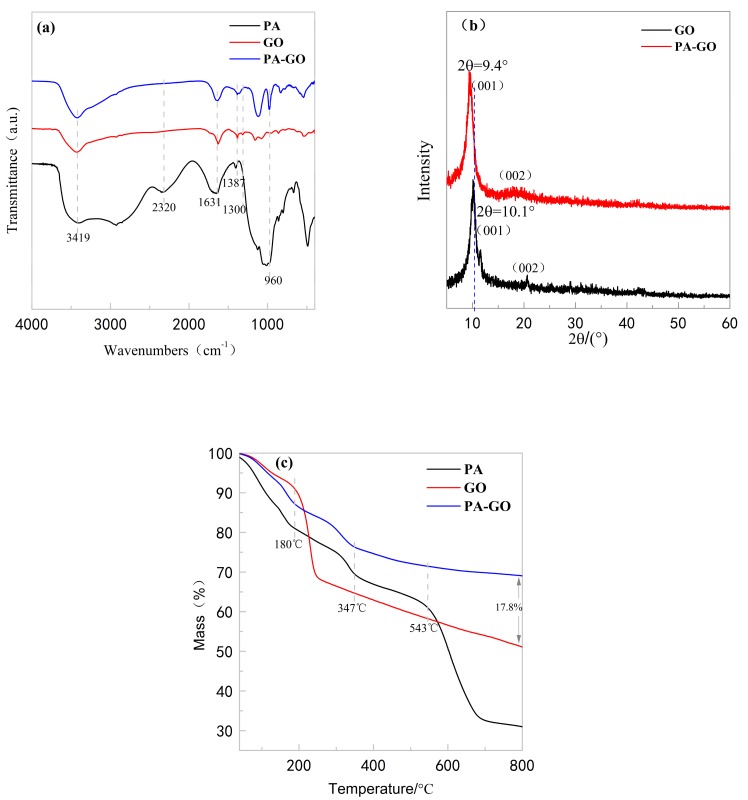
(**a**) FTIR spectra, (**b**) XRD patterns and (**c**) TGA of PA, GO, and PA-GO.

PA, GO, and PA-GO were characterized using FT-IR. The results are shown in Figure 2a, the absorption peaks at 3424 cm^−1^ (–OH), 1387 cm^−1^ (C=O), 1630 cm^−1^ (the stretching vibration absorption peak of carboxyl group), and 1300 cm^−1^ (C–O–C) are characteristic of GO [31]. The red solid line in Figure 2a is PA-GO. Compared with the line of GO, a new absorption peak appears at 960 cm^−1^ (C–O–P). In PA and GO, the absorption peak at 2338 cm^−1^ (P–OH) of PA and 1300 cm^−1^ (C–O–C) of GO disappear. This is mainly caused by the ring-opening reaction between the phosphate group in phytic acid (PA) and epoxy group in GO. The aforementioned results indicate the successful preparation of PA-GO.

The X-ray diffraction spectrum of GO and PA-GO are shown in Figure 2b, a sharp and a broad peak can be seen in the XRD pattern of GO at 2θ = 10.1° and 2θ = 20.7° which are attributed to (001) and (002) crystalline planes, respectively. The diffraction peak of GO was 10.1°, and the interlayer spacing of GO was 0.87 nm as calculated from (001) peak using Bragg’s equation [32] (nλ = 2dsinθ). The narrow peak of the PA-GO at approximately 9.4° corresponds to interlamellar spacing (0.94 nm) because some oxygen groups were present on the surface of the GO that led to the increase in interlamellar spacing (Confirm from FTIR analysis). These results indicate that PA is successfully inserted into the oxidation, leading to greater spacing between the GO layers.

The thermal decomposition behavior of PA, GO and PA-GO are shown in Figure 2c. The first stage of mass loss terminates at approximately 180 °C, which was due to the rejection of the adsorbed water from the interlayers of these materials. The comparable mass losses for GO and PA-GO were 8.0% and 11.9%, respectively, indicating that PA-GO has a stronger water absorption capacity than GO. The second stage of mass loss from 180 to 347 °C was attributed to the fact that the oxygen-containing groups on the surface of the sample are decomposed by heat and volatilized in the form of CO and CO_2_. However, at 800 °C, the residual mass of PA was 30.1%, while the residual mass of PA-GO (69.1%) was greater than that of GO (51.3%). This phenomenon was caused by the modification of PA.

**Figure 3:**
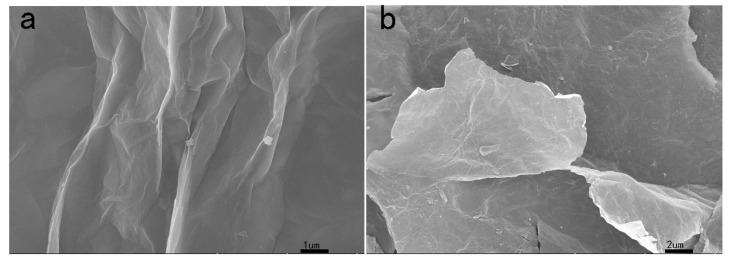
SEM of GO (**a**) and PA-GO (**b**).

Scanning electron microscope images of GO modified by GO and PA are shown in Figure 3. As shown in Figure 3a, the GO morphology is characterized by a layer of smooth folds. However, the morphology of modified graphene (PA-GO) in Figure 3b is significantly different from that of GO. It is clear from the PA-GO figure that more granular materials, small white particles, and more rough folds appear in the single sheet structure. These features are a further indication of phytic acid (PA) grafted on GO [27].

**Figure 4:**
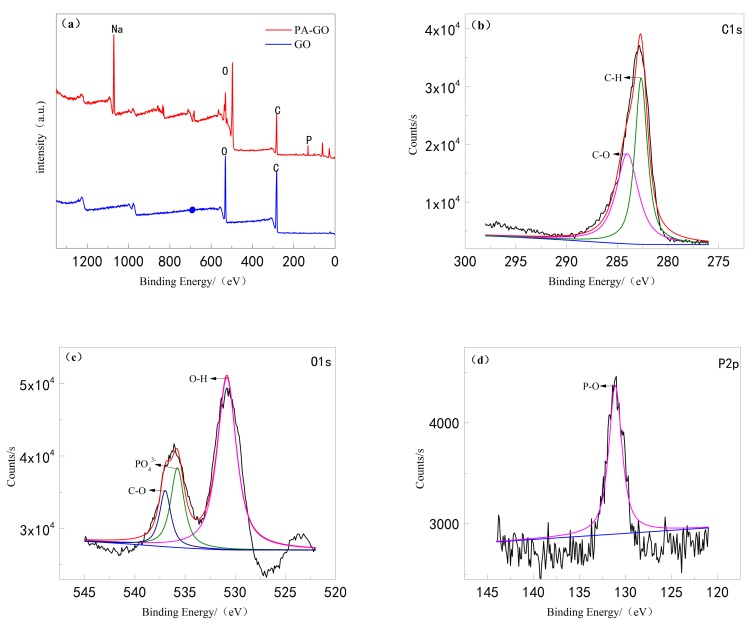
(**a**) XPS full spectra of GO and PA-GO. (**b**) C1s spectra of PA-GO. (**c**) O1s spectra of PA-GO. (**d**) P2p spectra of PA-GO.

The X-ray photoelectron spectrum analysis curve is shown in Figure 4. To further determine the preparation of PA-GO, X-ray photoelectron spectroscopy (XPS) was used to analyze the types and states of different elements on the surface of PA-GO. Figure 3a presents X-ray photoelectron spectroscopy for GO and PA-GO. Based on the wide spectrum in Figure 4a, several main characteristic peaks of GO and PA-GO are located at 1071, 529, 285, and 132 eV, corresponding to NaOH, O1s, C1s, and P2p signals, respectively. This finding is consistent with FTIR results. Since NaOH was used as the reaction solution in the synthesis process, Na elements were introduced. A phosphoric acid peak appeared at 130 eV in the PA-GO curve, while no phosphoric acid peak was detected in the GO sample. Therefore, the introduction of element P was evident. C1s spectral fitting analysis (Figure 4b) showed the presence of C–O (284.2 eV), C–C and C–H (282.7 eV) peaks [33]. The O1s spectral fitting analysis (Figure 4c) showed the characteristic peaks of oxygen in phosphate (535.2 eV), O–H group (531.7 eV), and C–O (536.2 eV) [34,35]. P2p spectral fitting analysis (Figure 4d) showed only one peak, namely, the absorption peak of P–O (131.2 eV) [36]. 

**Figure 5:**
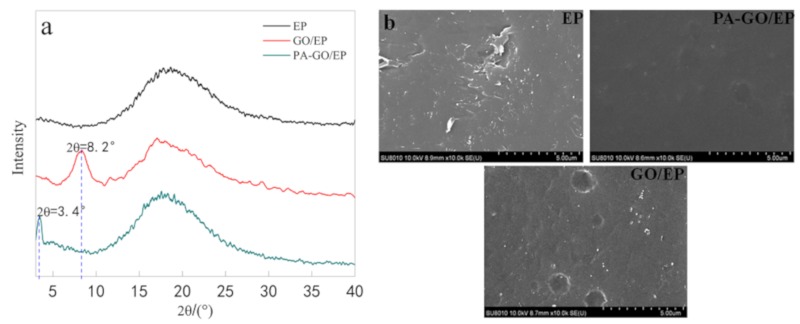
(**a**) XRD and (**b**) SEM of EP, GO/EP, and PA-GO/EP.

To evaluate the degree of intercalation of the GO and PA-GO nanosheets in the epoxy coatings, XRD analysis was employed, and the results are shown in Figure 5a. In Figure 5a, the broad peak was observed in the 2θ range of 14–25° in the EP sample was attributed to the scattering of amorphous structure of polymer chains [37]. Incorporation of GO nanosheets led to the appearance of a new broad peak at 2θ = 8.2°, exhibiting 1.07 nm interlayer space distance related to (001) diffraction of graphene oxide nanosheets. The d-spacing value of the GO nanosheets is 0.87 nm in Figure 2b. This result indicated the GO particles interlayer distance increased after inclusion into the epoxy matrix, revealing that partial nanosheets exfoliation resulted from epoxy chain diffusion into nanosheets. In the PA-GO/EP samples, a similar phenomenon was observed. In addition to the broad peak, another peak was observed at 2θ = 3.4°, and the corresponded d-spacing values were equal to 2.58 nm. However, the d-spacing values of GO/EP and PA-GO/EP were increased compared with that of the nanosheets GO and PA-GO. The higher d-spacing of the PA-GO/EP nanocomposite compared to the GO/EP sample is related to the dispersion improvement of the PA-doped GO nanosheets [38]. This finding is also evident in Figure 5b. This was potentially attributed to the presence of the PA-based nanoparticles in the intergalleries of the grapheme oxide layers. In other words, the PA grafted on the GO surface may decrease the π-π stacking interactions between the sp2 structure of GO nanosheets, causing better dispersion of nanopigments in the epoxy matrix.

### 3.2. Evaluation of Protective Performance 

**Figure 6:**
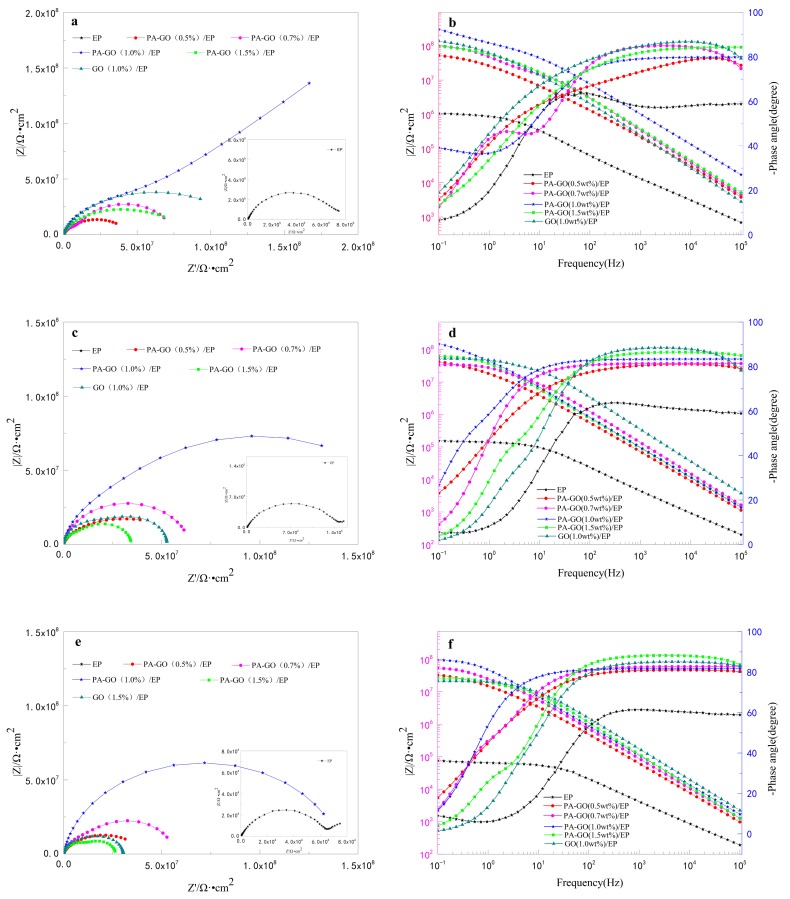
EIS plots of different coating/steel systems under 3.5% NaCl solution at various time intervals: (**a**,**b**) 48 h, (**c**,**d**) 540 h, and (**e**,**f**) 1080 h.

EIS measurements for the coated mild steel specimens were performed by immersing them in 3.5 wt.% NaCl solution for a total of 1080 h. Figure 6 records the Nyquist plot and bode plot of coating with different fillers at different immersion times. The Nyquist curve mainly reflects the law of the resistance value of anticorrosive coating changing with time. The radius of the curve can directly reflect the impedance of the coating, which indicates the shielding effect of the coating on the metal surface. During the curing process of the coating, micropores will be generated on its surface. Under the long-term immersion of NaCl solution, corrosive ions in the brine will pass through the micropores, thus passing through the coating and directly contacting with the substrate, and then chemical reactions will occur on the substrate surface, resulting in corrosion on the surface of the body [39]. For the bode plot, the impedance value of low frequency region (|Z|_0.1_) represents the strength of electrochemical corrosion resistance of the coating [40].

In Figure 6, at the beginning of immersion, the coatings with fillers in Figure 6a showed great resistance. Combined with the bode plot in Figure 6b, all coatings approximately showed only one time constant. Even if the signal appeared as a one time constant, more than a one time constant is present, and the time constants overlap enough to mask true nature of the system. The coating impedance of the low-frequency region exhibits the following order: PA-GO (1.0 wt.%)/EP > GO (1.0 wt.%)/EP > PA-GO (0.7 wt.%)/EP > PA-GO (1.5 wt.%)/EP > PA-GO (0.5 wt.%)/EP > EP. In this stage, the organic coating can completely cut off corrosion particles and prevent the occurrence of corrosion. It can also be seen from the figure that the addition of layered filler (GO or PA-GO) helps to improve the corrosion resistance of the coating. As the amount added increases, impedance values first increase then decrease. This finding indicates that a small amount of filler is not sufficient to make up for the defect of epoxy resin, and the addition of too much material will cause the filler to reaggregate in epoxy resin, resulting in new defects and decreased coating resistance. Therefore, PA-GO (1.0 wt.%)/EP shows excellent performance. The impedance value of GO (1.0 wt.%)/EP is significantly lower than that of PA-GO (1.0 wt.%)/EP, possibly due to the poor compatibility between GO and epoxy resin.

**Figure 7:**
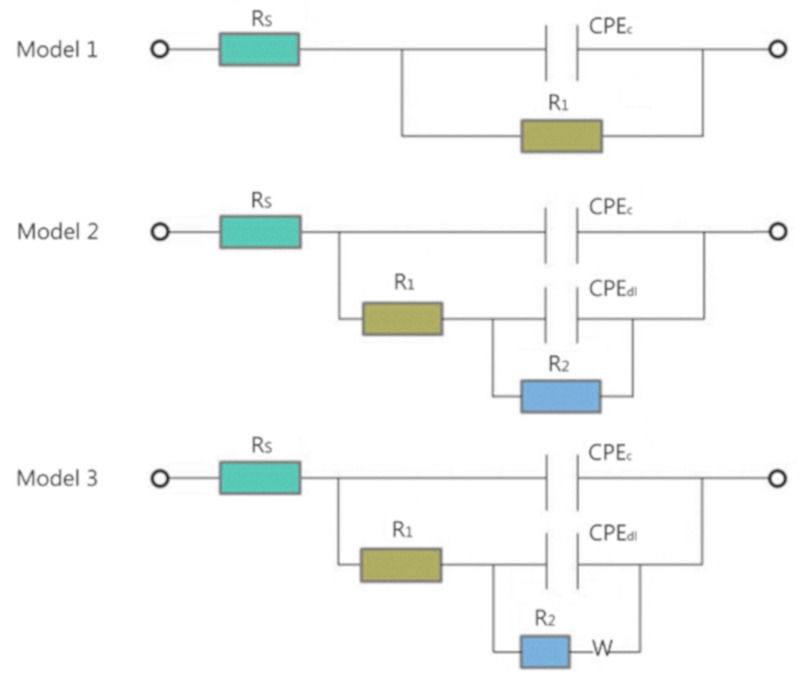
Equivalent electrical circuits proposed for the coatings.

As the immersion time increases, the above phenomenon is still evident. However, as shown in Figure 6b,c, the impedance of the coating performance exhibited a reducing trend due to the absorption of corrosive medium and the penetration of corrosive particles through the defects and holes in the coating surface. A new diffusion field at the low frequencies appears around the EP-coated steel electrode. At this time point, the equivalent circuit contains a Warburg impedance element (W) (Figure 7 Model 3) because the corrosion of the EP-coated steel electrode is mainly affected by the diffusion effect of coating or coating-substrate interface at the low-frequency band. The other coatings still correspond to the equivalent circuit model 2 in Figure 7. As the immersion time is extended to 1080 h, a continuous downward trend appeared in Figure 6c,e. At this time point, corrosion is further accelerated by the diffusion of corrosion particles in the coating or coating-substrate interface. In addition to EP, the equivalent circuit of model 2 in Figure 7 was still applicable. To better compare the influence of PA-GO and GO on the anti-corrosion performance of waterborne epoxy resin, this paper next focuses on the comparison of the difference between samples with the added filler of 1.0 wt.% and EP.

Equivalent circuits:

Model 1: R_p_ = R_1_ (1)

Model 2: R_p_ = R_1_ + R_2_ (2)

Model 3: R_p_ = R_1_ + R_2_ (3)

Here, R_p_ represents the polarization resistance, R_s_ represents the solution resistance, W represents the diffusion resistance, R_1_ and R_2_ represent the resistors in the circuits, and CPE represents the constant phase element in the circuits [20,41]. W appears because the corrosive ions are transported in the solution via diffusion in this process.

**Figure 8:**
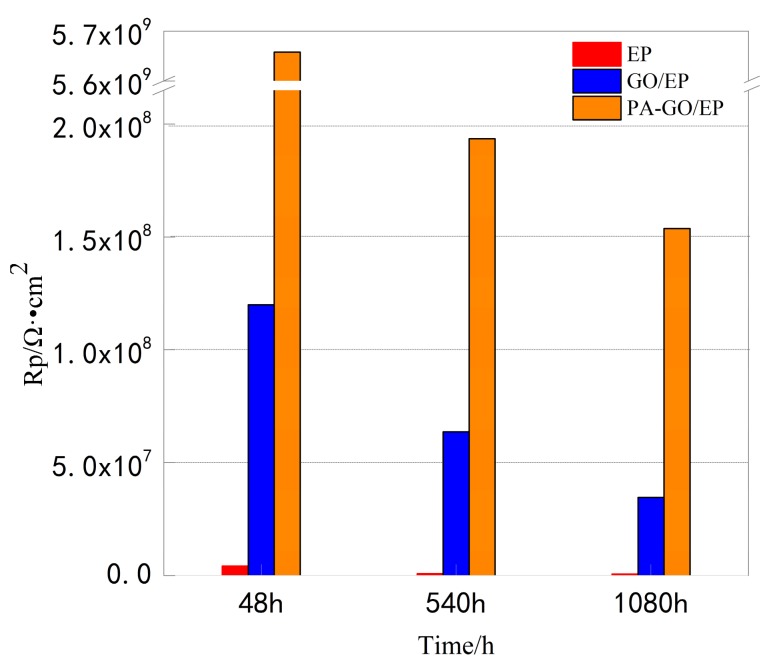
Polarization resistance of the three coatings at 48, 540, and 1080 h of immersion in 3.5 wt.% NaCl solution.

Three equivalent circuits to match the EIS models were established by the immersion test in Figure 6, and the fitting results are shown in Table 2. Because different models are used to adapt to different scenarios, it is impossible to compare fitting results directly. Therefore, polarization resistance (R_p_), which is defined as the real impedance difference between the zero frequency and Nyquist diagram solution resistance [42,43], was calculated for comparison. The calculation of the polarization resistance in the 3.5 wt.% NaCl solution under the sample dipping times of 48, 540, and 1080 h is shown in Figure 8. Initially, the PA-GO (1.0 wt.%)/EP coating showed the largest R_p_ value of 5.646 × 10^9^ Ω∙cm^2^. The R_p_ value for the EP and GO (1.0 wt.%)/EP coating was relatively low at 4.149 × 10^6^ Ω∙cm^2^ and 1.088 × 10^8^ Ω∙cm^2^, respectively. The samples were immersed in NaCl solution for 540 h and 1080 h, and this trend remained evident. After the dip corrosion test, the polarization resistance of all samples decreased, but PA-GO/EP still showed better corrosion resistance than EP and GO/EP. In summary, the current research results confirm that: (1) the layered structure of GO can improve the barrier performance of epoxy coating and the anti-corrosion performance of coating; (2) the insertion of PA in GO can improve the corrosion resistance of GO in the coating.

**Figure 9:**
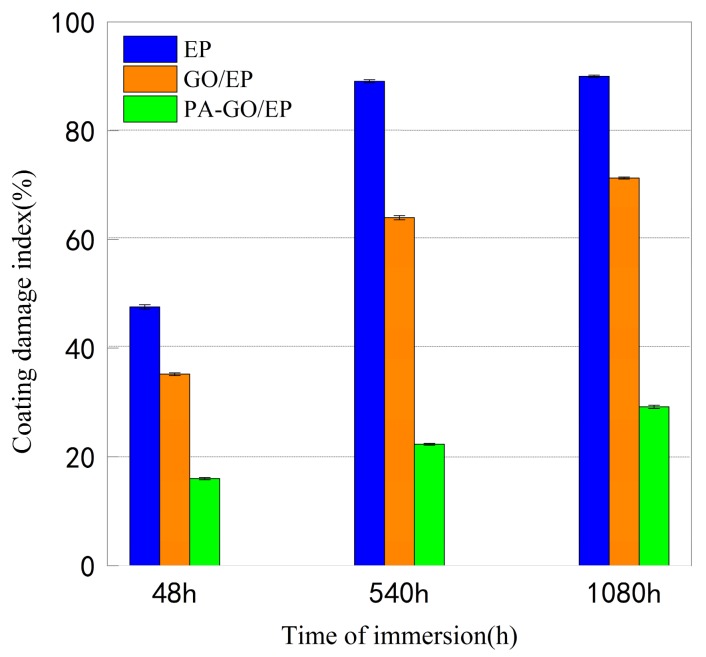
Coating damage index values for EP, GO/EP, and PA-GO/EP samples after 48, 540, and 1080 h immersion in 3.5 wt.% NaCl solution.

The coating damage index (CDI) is a parameter used to describe the corrosion resistance behavior of coated metals [44]. Figure 9 shows the changes of CDI of EP, GO/EP, and PA-GO/EP samples over time. Due to the diffusion of water, oxygen, and corrosive ions into the film, the coating damage index of all samples increased as the immersion time increased. The epoxy coating damage was reduced to some extent in the presence of GO nanosheets. In corrosive environments, the electrolyte penetrates through the coating in a capillary manner, gradually causing the pores in the coating to expand and accelerating the destruction of the coating. As noted in Figure 8, compared with GO/EP samples, the addition of PA-GO is more effective in delaying such expansion and reducing the coating damage index. This is mainly due to the phytic acid-modified graphene oxide, which improves pigment nanoparticles dispersed in the organic coating, increases the compatibility of the filler and matrix, and decreases the coating damage index.

**Figure 10:**
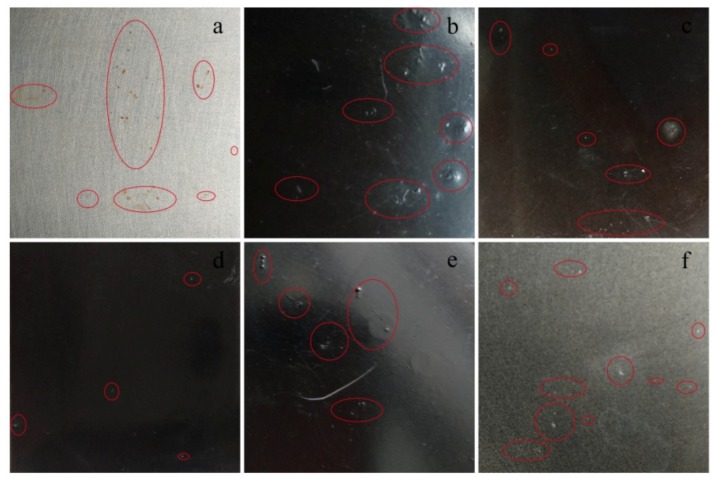
Surface morphology of the coatings after the salt spray test for 600 h: (**a**) neat EP; (**b**) PA-GO (0.5 wt.%)/EP; (**c**) PA-GO (0.7 wt.%)/EP; (**d**) PA-GO (1.0 wt.%)/EP; (**e**) PA-GO (1.5 wt.%)/EP; and (**f**) GO (1.0 wt.%)/EP.

Figure 10 shows the conditions of waterborne epoxy coatings with different filler contents after a 600 h salt spray test. The results showed that the coating surfaces of a–f had different degrees of blistering and pitting phenomena, indicating that the salt spray has already had corrosion particles infiltrating into the coating through microholes during the process of spraying, which causes the pitting phenomenon of the steel sheet surface substrate. Among them, the corrosion in Figure 10a is the most serious, while the corrosion spots of samples in Figure 10d are relatively minimal, indicating that the addition of PA-GO and GO filler can help plug up the micropore channels generated in the curing process of epoxy coating, thereby improving the anti-corrosion performance of the coating. When the addition amount is 1.0 wt.%, the compatibility between the coating base material and the filler is optimal, so the anti-corrosion performance is the best. However, there are many corrosion points on the surface of sample samples of the component in Figure 10e, which indicates that an excessive amount of filler aggregates in the water-based matrix. This feature promotes the generation of new microcracks, causing corrosion particles to enter into the coating quickly, accelerating corrosion, and thus reducing the anti-corrosion performance of the coating. Comparing samples in Figure 10d,f, the results are consistent with the electrochemical data. Specifically, phytic acid-modified GO exhibits improved corrosion resistance in the coating. This is mainly due to the better dispersion of PA-GO, which is consistent with the results in Figure 5.

## 4. Conclusions

The anticorrosion ability of the waterborne epoxy (EP) coating can be improved by adding layered fillers. In this work, we demonstrated that: (i) the modification of GO with PA is successful, and (ii) Compared with EP and GO/EP, the corrosion resistance of PA-GO (1.0wt.%)/EP in NaCl solution was significantly improved by EIS (with 3.5 wt.% NaCl solution) and the salt spray test (with 5.0 wt.% NaCl solution). This feature is mainly related to the improved dispersion of PA-GO in EP.

## Figures and Tables

**Table 1:** Composite coating formulation with phytic acid-doped graphene oxide (PA-GO) and GO.

Samples	Waterborne Epoxy Resin (g)	Pigment (g)	Curing Agent (g)	Deionized Water (g)
Neat EP	20	-	8	6
GO (1.0 wt.%)-EP	20	0.2	8	6
PA-GO (0.5 wt.%)-EP	20	0.1	8	6
PA-GO (0.7 wt.%)-EP	20	0.14	8	6
PA-GO (1.0 wt.%)-EP	20	0.2	8	6
PA-GO (1.5 wt.%)-EP	20	0.3	8	6

**Table 2:** Parameter values obtained from the simulation circuit for the coatings immersed in 3.5 wt.% NaCl solution up to 1080 h.

Samples	Time (h)	R_s_ (Ω·cm^2^)	R_1_ (Ω·cm^2^)	CPE_c_ (F/cm^2^)	R_2_ (Ω·cm^2^)	CPE_dl_ (F/cm^2)^	W (Ω·cm^2^)	Model
EP	48	7.864 × 10^1^	5.397 × 10^6^	1.576 × 10^−7^	7.523 × 10^5^	1.003 × 10^−8^	-	2
	540	3.93 × 10^1^	6.264 × 10^5^	4.207 × 10^−7^	1.699 × 10^5^	2.265 × 10^−4^	8.956 × 10^−16^	3
	1080	3.856 × 10^1^	2.960 × 10^3^	1.547 × 10^−7^	6.149 × 10^5^	9.825 × 10^−8^	8.873 × 10^−5^	3
GO (1.0 wt.%)/EP	48	2.724 × 10^2^	1.088 × 10^8^	1.012 × 10^−9^	4.140 × 10^4^	5.786 × 10^−9^	-	2
	540	8.210 × 10^2^	2.916 × 10^7^	4.485 × 10^−10^	2.330 × 10^7^	2.736 × 10^−9^	-	2
	1080	4.514 × 10^2^	7.107 × 10^6^	7.430 × 10^−10^	1.634 × 10^7^	9.620 × 10^−10^	-	2
PA-GO (1.0 wt.%)/EP	48	8.047 × 10^1^	5.646 × 10^9^	5.834 × 10^−10^	-	-	-	1
	540	1 × 10^2^	8.398 × 10^7^	3.093 × 10^−9^	9.805 × 10^7^	6.177 × 10^−9^	-	2
	1080	1 × 10^3^	1.422 × 10^8^	2.289 × 10^−9^	3.896 × 10^4^	9.446 × 10^−10^	-	2
